# The draft genome sequence of cork oak

**DOI:** 10.1038/sdata.2018.69

**Published:** 2018-05-22

**Authors:** António Marcos Ramos, Ana Usié, Pedro Barbosa, Pedro M. Barros, Tiago Capote, Inês Chaves, Fernanda Simões, Isabl Abreu, Isabel Carrasquinho, Carlos Faro, Joana B. Guimarães, Diogo Mendonça, Filomena Nóbrega, Leandra Rodrigues, Nelson J. M. Saibo, Maria Carolina Varela, Conceição Egas, José Matos, Célia M. Miguel, M. Margarida Oliveira, Cândido P. Ricardo, Sónia Gonçalves

**Affiliations:** 1 Centro de Biotecnologia Agrícola e Agro-alimentar do Alentejo (CEBAL) / Instituto Politécnico de Beja (IPBeja), Beja 7801-908, Portugal; 2 Instituto de Ciências Agrárias e Ambientais Mediterrânicas (ICAAM), Universidade de Évora, Évora 7006-554, Portugal; 3 Instituto de Tecnologia Química e Biológica António Xavier, Universidade Nova de Lisboa (ITQB NOVA), Oeiras 2780-157, Portugal; 4 Instituto de Biologia Experimental e Tecnológica (iBET), Oeiras 2780-157, Portugal; 5 INIAV - Instituto Nacional de Investigação Agrária e Veterinária, Oeiras 2780-157, Portugal; 6 Biocant – Associação de Transferência de Biotecnologia, Cantanhede 3060-197, Portugal; 7 Centro de Neurociências e Biologia Celular, Universidade de Coimbra, Coimbra 3004-504, Portugal; 8 Centre for Ecology, Evolution and Environmental Changes - cE3c, Faculdade de Ciências, Universidade de Lisboa, Lisboa 1749-016, Portugal; 9 Biosystems & Integrative Sciences Institute, Faculdade de Ciências, Universidade de Lisboa (FCUL), Lisboa 1749-016, Portugal

**Keywords:** Plant sciences, Genome, Computational biology and bioinformatics, DNA sequencing

## Abstract

Cork oak (*Quercus suber*) is native to southwest Europe and northwest Africa where it plays a crucial environmental and economical role. To tackle the cork oak production and industrial challenges, advanced research is imperative but dependent on the availability of a sequenced genome. To address this, we produced the first draft version of the cork oak genome. We followed a *de novo* assembly strategy based on high-throughput sequence data, which generated a draft genome comprising 23,347 scaffolds and 953.3 Mb in size. A total of 79,752 genes and 83,814 transcripts were predicted, including 33,658 high-confidence genes. An InterPro signature assignment was detected for 69,218 transcripts, which represented 82.6% of the total. Validation studies demonstrated the genome assembly and annotation completeness and highlighted the usefulness of the draft genome for read mapping of high-throughput sequence data generated using different protocols. All data generated is available through the public databases where it was deposited, being therefore ready to use by the academic and industry communities working on cork oak and/or related species.

## Background & Summary

*Quercus suber* is an evergreen tree, commonly known as cork oak, which is native to the western Mediterranean Basin, especially southwest Europe, where it occurs in the coastal regions. Cork oak has the rare characteristic of producing a continuous and renewable cork layer, which has fine physical and chemical properties that make it highly profitable for industrial uses.

The cork oak is the basis of an ecological system, known as “montado” that is unique in the world, contributing to the survival of many native species of fauna and flora and preventing desertification in vulnerable areas. Over the last few years, a decline in cork oak populations has been observed, eventually as a result of agriculture intensification, biotic stresses, fires and climate changes. If this trend is not stopped, serious negative impacts may arise at the ecosystem, social and economic levels.

The availability of a fully sequenced and annotated genome is essential to support many of the studies needed to answer fundamental questions about cork oak biology and interactions with the environment, and about cork formation/production, as well as to develop the tools required to address the problems presently affecting this species. In fact, despite the economic relevance of cork production, no genetic breeding/selection schemes have so far been established in this species, thus compromising the development of genetically superior trees, able to produce high quality cork, displaying enhanced resistance to the (a)biotic stress factors they are exposed to and with potential to adapt to new forestry management practices that may be implemented in the future.

The development of genetic markers in cork oak is an extremely important field, which will greatly benefit from the availability of a reference genome. For example, cork quality, perhaps the most important trait in cork oak production, cannot be properly evaluated before at least 40 years of growth, due to the specificities of the production cycle. This extremely long cycle represents a textbook scenario in which genetic markers associated with better cork quality can be highly beneficial. The same reasoning can be applied to other relevant traits, such as resistance to drought, diseases, pests, or developmental modifications important to face climate changes.

A comprehensive characterization of the cork oak transcriptome was previously produced^1^, which increased the amount of genomic resources available for cork oak and allowed transcriptomic analyses of several biological systems, such as the response to ectomycorrhizal symbiosis establishment^2^, response to drought^3^ and flower and fruit development^4,5^.

To assist and enhance the development of the solutions needed for the future of cork oak production we have generated a draft version of the cork oak genome (Data Citation 1 and Data Citation 2).

The genome size of cork oak, a diploid (2n=24) species, was estimated, using flow cytometry, to be 934 Mb^6^. In the present study we used a combination of Paired-End (PE) and Mate-Pair (MP) libraries sequenced using the Illumina platform to generate a draft genome assembly with an estimated genome size of 953.3 Mb, which is a very close match to the previous estimate. The bioinformatics pipeline involved a *de novo* genome assembly step, followed by scaffolding, gap filling and removal of heterozygous regions. The cork oak draft genome is distributed over 23,344 scaffolds, even though the vast majority of the assembly is represented in a considerable smaller number of larger scaffolds (approximately 94.6% of the assembled genome present in the 4,730 scaffolds longer than 10,000 bp).

The structural annotation of the genome yielded 79,752 genes, with complete open reading frames, and 83,814 transcripts. The number of transcripts with a valid functional annotation varied with the database used, and the maximum number was 69,218, when searching against InterPro signatures, which represented 82.6% of the total. Finally, using a validation approach based on the RNA-Seq data available for five cork oak tissues, a total of 33,658 predicted genes could be confirmed and classified as high confidence genes, since they presented assembled transcripts within the genome annotation coordinates.

## Methods

### Selection of the target individual for genome sequencing

Interspecific hybridization is a widespread phenomenon in plants, which also occurs among species of the *Quercus* genus. In fact, hybridization between *Quercus suber* and *Quercus Ilex*, mainly *Quercus ilex* subsp. *rotundifolia*, another species widely distributed in the Mediterranean region, is fairly common, since both are found in mixed stands where they cohabit. Even though some of these hybrids maintain the ability to produce cork, only a pure cork oak tree can produce high quality cork. Thus, in order to select the tree to sequence, a total of 28 trees, derived from four different locations, were selected, based on historical records that demonstrated their ability to produce very high quality cork. These individuals fulfilled an estimated median age of more than 50 years, were at least 50 meters apart (to reduce chances of selecting possibly related individuals), and were not planted in rows, to ensure that they were naturally grown trees. Leaves were collected from these trees for DNA extraction. Then, all individuals were genotyped with 16 microsatellite markers, to estimate the degree of homozygosity, since a higher level is preferable for a genome sequencing project. The microsatellite markers used were selected from available literature, and included markers developed in *Quercus mongolica*^7^, *Quercus petraea*^8^, *Quercus robur*^9^, *Quercus macrocarpa*^10^, *Quercus myrsinifolia*^11^, *Castanea sativa*^12^ and two additional markers developed for cork oak at CEBAL and INIAV (published here). This information is summarized in Table 1.

Multiplex PCR amplification was conducted in a GeneAmp PCR system 2700 (Applied Biosystems) and PCR products were analyzed on an ABI Prism 310 capillary electrophoresis system (Applied Biosystems). Size alignment and quality control were analyzed on Genescan 3.7 (Applied Biosystems). The number of homozygous loci per tree ranged from four to nine, the latter being observed in three cork oaks located at Herdade dos Leitões (Montargil, Ponte de Sor, Portugal). From these three individuals the HL8 cork oak was considered the most robust tree and selected for genome sequencing.

### Extraction of nucleic acids and high-throughput sequencing

Leaves collected from the HL8 cork oak tree were used for DNA extraction. To reduce contamination with chloroplast and mitochondrial DNA nuclei were first isolated using the CelLytic™ PN Isolation/Extraction Kit (Sigma) and nuclear DNA was then extracted using the innuPREP Plant DNA Kit (AnalytiK Jena).

All Illumina DNA sequencing was performed at the Beijing Genomics Institute (BGI), and included a combination of PE and MP libraries (Data Citation 2), sequenced using different Illumina sequencers (Table 2).

A total of 10,560,988,448 reads were generated. The raw reads were filtered by removing adapter sequences and reads containing undetermined nucleotides (N’s). The reads that remained in the dataset were further processed with Sickle^13^, which trimmed/removed the low quality reads, using as parameters minimum quality (equal or larger than 20) and minimum length (80% of the read length). To remove reads derived from the chloroplast and mitochondrial genomes the high quality reads were mapped, with BWA using the BWA-mem algorithm^14^, against eight mitochondrial and eight chloroplast genomes. These genomes were selected from the species more closely related to cork oak for which these organelle genomes were available at the time this step was executed. The NCBI Reference Sequence numbers for each of these genomes are indicated in Table 3.

**Figure 1:**
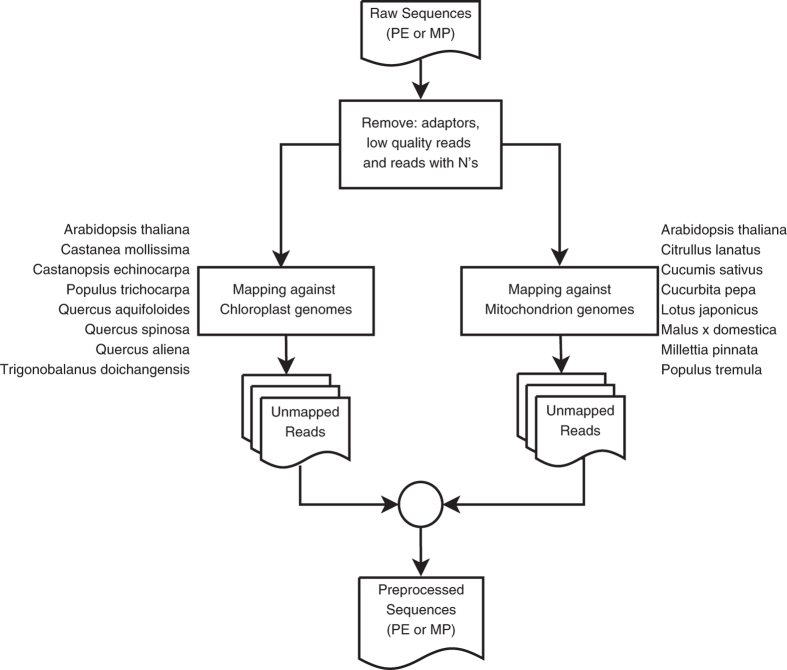
Illumina DNA sequence data pre-processing workflow. The pipeline included removal of low quality reads, as well as reads containing adapter sequences and undetermined nucleotides. The reads that remained were subsequently mapped to a set of chloroplast and mitochondrion genomes to remove the reads derived from these plastid genomes.

The reads that did not map against any of the 16 genomes were kept and formed the set of Illumina DNA data that was used for all downstream analyses, which comprised 8,544,112,336 reads (80.9% of the initial number of reads). Fig. 1 provides a detailed description of the pre-processing workflow.

A total of five tissues were sampled from the HL8 cork oak tree, in order to generate the RNA-Seq data (Data Citation 2) needed for the genome annotation stage (Table 4). These tissues included pollen, leaf, xylem, inner bark and phellem. Total RNA was extracted from all tissues using the Plant/fungi Total RNA purification kit (Norgen). All Illumina RNA-Sequencing was performed using the HiSeq 4000 system, with a read length of 100 nucleotides and a paired-end sequencing protocol.

A total of 1,530,447,601 RNA-Seq reads were produced. These reads were further processed using a similar approach to the one described for the DNA sequence data, which included the application of the same procedures for the removal of reads with adapters or undetermined nucleotides and trimming of the reads. The number of reads kept amounted to 1,438,136,157, which represented 94% of the initial number of reads.

### Cork oak genome properties

**Figure 2:**
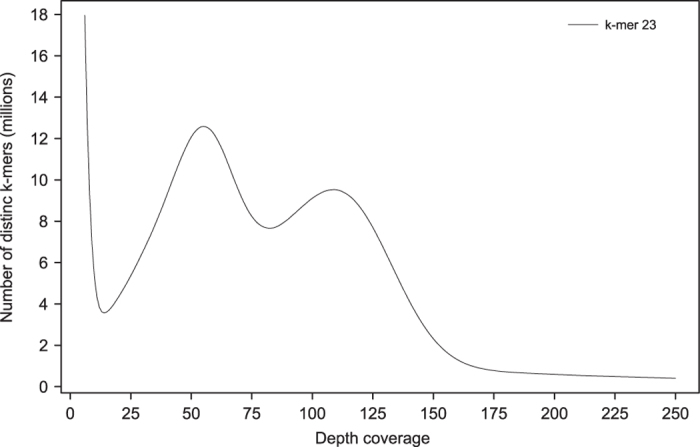
K-mer distribution used for the estimation of genome size. The distribution was determined with Jellyfish using a k-mer size of 23.

A k-mer analysis of the PE libraries with read length of 100 bp was performed to characterize the genome sequence using Jellyfish^15^ with a k length of 23. The k-mer spectrum followed a binomial distribution expected for a heterozygous diploid genome (Fig. 2). The homozygous peak (right peak) was observed at 109x, while the heterozygous peak (left peak) was observed at 55x. There were noticeably more heterozygous k-mers than homozygous k-mers, which indicated a high level of heterozygosity. The diploid genome size was estimated following the approach described by Sork and colleagues^16^, which included (1) dividing all k-mers under the haploid peak, except the error k-mers (all k-mers represented with a single read), by the haploid coverage depth, (2) dividing all k-mers counted between the haploid coverage peak +1 and the diploid coverage peak by the diploid coverage depth, and (3) summing those values. The estimated cork oak genome size based on sequence data was 968.7 Mb, a value that slightly exceeds the genome size estimated by flow cytometry (934 Mb)^6^.

Additionally, GenomeScope^17^ was also used to estimate genome size and heterozygosity, using the k-mer distribution determined with a k-mer size of 23, which provided estimates of 1.06 Gb and 1.62% for genome size and heterozygosity, respectively.

### Genome assembly

The Illumina reads were used to generate the genome assembly, a process that was performed in two stages, due to the different read length of the PE libraries. Hence, two independent genome assemblies were produced, one for each type of read length, using Ray^18^, the assembler that was selected because it provided the best results (less contigs, larger genome assembly, larger N50) after testing several assembly tools. The k-mer sizes used in the assembly were 81 and 121, for the PE libraries with a read length of 100 bp and 150 bp, respectively. The best k-mer size was estimated with SGA preqc^19^.

The contigs that resulted from each assembly were integrated into a single assembly using GARM^20^, in order to obtain a set of contigs common to both individual assemblies and identify the unique contigs from each assembly.

The latter set of contigs was used as the reference on which all PE reads were mapped using BWA with the BWA-mem algorithm^14^, in order to differentiate the contigs representing valid genome regions from the erroneous contigs possibly derived from the assembly step. The mapping results were analyzed for coverage using BEDtools^21^. We considered as valid all the contigs which displayed a minimum length of 1,000 bp, at least 95% of the length covered with mapped reads, and that 95% of the regions with mapped reads had a minimum coverage of 50x. The contigs that passed these filters were added to the set of contigs common to both assemblies, which formed the assembly used to proceed with scaffolding of the genome. The total number of contigs that were used as the reference for the scaffolding step was 168,041, and comprised 939,042,321 bp of sequence (Table 5).

The mate-pair read dataset was mapped to the assembly using BWA with the BWA-mem algorithm^14^. The mapped reads were filtered for quality (minimum quality score of 10) and subsequently used for scaffolding the genome using BESST^22^, using the mate-pair libraries in ascending order relative to their respective insert size. In order to reduce the number of gaps two rounds of gap closing were performed using SOAP de novo Gap Closer^23^,using in each round a different set of two PE libraries from all available insert sizes (170 bp, 300 bp, 500 bp, 800 bp). Given the high heterozygosity of the cork oak genome, a final step targeting the removal of possible alternative heterozygous scaffolds was performed, using Redundans^24^, on an assembly that contained 44,287 scaffolds and comprised 1,000,388,124 bp. A total of 47.1 Mb of assembled sequence was removed, which represented a 4.7% decrease, while 20,943 scaffolds were discarded, a decrease of 47.3%. These results represent an approximate haplotype duplication level for the cork oak draft genome sequence.

The final assembly of the cork oak draft genome contained 23,344 scaffolds greater than 1,000 bp, which represented an assembly length of 953.3 Mb (Table 6). The percentage of undetermined nucleotides was 2%. The vast majority of the assembled genome was contained in the larger scaffolds. For instance, approximately 94.6% of the assembled genome was present in the 4,730 scaffolds longer than 10,000 bp. Similarly, the 2,022 scaffolds with a minimum length of 100 Kb contained 823.7 Mb of the genome, which represented 86.4%. These were clear indications that the majority of the fragmentation observed in the draft genome was related to shorter scaffolds, which may be due to the high heterozygosity and unsolved repeats of the cork oak genome. The N50 observed was 465.2 Kb, while the longest scaffold was 2,284,287 bp in length.

### Genome annotation

Augustus^25^ was employed to predict gene models in cork oak. To achieve best performance, species-specific parameters were generated by training Augustus with a subset of cork oak genes. To generate this subset, Maker^26^ was run with transcripts obtained from the five HL8 RNA-Seq tissues assembled with a Star^27^ and StringTie^28^ pipeline and protein sequences from *Arabidopsis thaliana*. The run with Maker was stopped when a reasonable number of genes had been predicted, which resulted in a total of 1,268 genes selected to train Augustus. In order to maximize annotation accuracy several sources of external hints were produced to enhance the Augustus gene predictions. Initially, a custom repeat library was generated with RepeatModeler^29^, which was then used to perform the repeat masking of the genome with RepeatMasker^30^. Augustus repeat hints were obtained based on the RepeatMasker output. In addition, protein hints were generated based on the genome coordinates where CEGMA^31^ conserved proteins mapped (please check the section “Technical Validation”). Exonic and intronic hints were produced based on the STAR mappings of the HL8 RNA-Seq libraries from four tissues (leaf, xylem, inner bark, phellem) using Augustus utilities (bam2hints, wig2hints). Finally, Augustus was run by adding the option to also report alternative transcripts when they were suggested by hints.

The number of genes annotated for the cork oak draft genome was 79,752. The final list included only the genes for which a start and stop codon was detected. Additionally, a total of 83,814 transcripts were identified, an indication of the presence of alternative splicing events in the cork oak transcriptome.

Functional annotation was performed for the set of 83,814 transcripts using different sources of information. Detection of homologies in the predicted gene models was performed using BLASTP against the databases NCBI-nr and Swiss-Prot^32^. Additionally, eggNOG-mapper^33^ was used to assign orthologies based on precomputed phylogenies of Viridiplantae organisms from the eggNOG database^34^. Last, InterProscan^35^ was used to detect protein domains, Gene Ontology terms and KEGG mappings from the InterPro database^36^.

The percentage of functionally annotated transcripts varied from 55.6% to 82.5%, for the SwissProt and InterPro databases, respectively (Table 7). The percentage of transcripts with a valid functional annotation is highly dependent on the information deposited in the publicly available databases used in this type of analyses. Moreover, considering that cork oak is a species with unique biological features, it is also possible that some genes unique to cork oak were identified. Consequently, these genes did not have any matches in the databases used, and the characterization of their function will require additional studies specifically targeting this goal. Overall, a total of 40,599 transcripts (37,724 genes) were annotated in all databases, which is a promising number, especially when considering that the SwissProt database contains manually curated protein sequences.

The percentage of the genome covered by repeat elements was 11.96%, for a total of 113,971,428 bp of the 953.3 Mb contained in the assembled cork oak draft genome (934.2 Mb after excluding the regions with Ns). These results were obtained running RepeatMasker using the eudicotyledons subset of RepBase, and are summarized in Table 8. Matches for retroelements accounted for 7.59%, while the percentage of DNA transposons and simple repeats was 0.74 and 2.72, respectively. These results are quite similar to the ones obtained for the valley oak genome^16^, and lower than the pedunculated oak genome, which appears to contain approximately 33% of repetitive elements^37^.

### Code Availability

The execution of this work involved using many software tools, whose versions, settings and parameters are described below.

**1) Sickle**: minimum quality (equal or larger than 20); minimum length (80% of the read length); **2) BWA**: version 0.7.15, default parameters; **3) Jellyfish**: version 2.2.6, k-mer size of 23; **4) GenomeScope**: parameters used were k-mer length 23; read length 80; maximum k-mer coverage 1000; **5) Ray**: version 2.3.1, default parameters, k-mer size of 81 for the assembly with the PE100 reads, k-mer size of 121 for the assembly with the PE150 reads; **6) SGA**: version 0.10.14, default parameters; **7) GARM**: version 0.7.5, default parameters; **8) BEDtools**: version 2.25.0; **9) BESST**: version 2.2.5, default parameters; **10) Gap Closer**: version 1.12, parameters used were -l 150, in configFile: asm_flags=4; PE100 lib: rd_len_cutoff=90, pair_num_cutoff=7, map_len=45; PE150 libs: rd_len_cutoff=140, pair_num_cutoff=10, map_len=50; **11) Redundans**: version 0.13a, with default parameters for homology, --nogapclosing, --noscaffolding, --norearrangements; **12) Augustus**: version 3.2.2, parameters: (--protein=on --codingseq=on --cds=on --introns=on -genemodel=partial --softmasking=1 --UTR=off --alternatives-from-evidence=true --uniqueGeneId=true; **13) Maker**: version 3.0.0 beta, parameters: est=merged transcripts from stringtie assemblies for 5 tissues, protein=arabidopsis_proteins, rmlib=repeatModelerOutput, est2genome=1, protein2genome=1; run was stopped when 2,574 scaffolds had been scanned, which predicted 2,394 genes genes, subsequently used to train Augustus; **14) Star**: version 2.5.2b, parameters: --outSAMattributes All --outFilterIntronMotifs RemoveNoncanonical --outWigType wiggle --outSAMtype BAM SortedByCoordinate --chimSegmentMin 20 --outReadsUnmapped Fastx --outFilterScoreMinOverLread 0.4 --outFilterMatchNminOverLread 0.4 --outFilterMatchNmin 60; **15) StringTie**: version 1.3.1b; parameters for first transcriptome assemblies for maker: default parameters; merge (samples per tissue and reference per tissue: -F 0.5 -T 0.5 -i); transcriptome assemblies for annotation validation: -c 5 -j 1.5 -a 20 -m 400 -f 0.2; **16) RepeatModeler**: RepeatModeler-open-1.0.8; **17) RepeatMasker**: RepeatMasker-open-4.0.6 (with specific specific cork oak library generated with repeatModeler using the scaffolds>5000 bp); **18) CEGMA**: version2.5; **19) Blastp**: version 2.6.0; **20) NCBI-nr**: we used the subset of green plant proteins extracted using taxonomic ID 33090, downloaded on 11th January 2017, for a total of 7,903,686 entries; **21) SwissProt**: we used the database version downloaded on 15th March 2017, for a total 593,119 entries, including isoforms; **22) eggNOG mapper**: version 0.12.7; **23) eggnog**: version 4.5; **24) InterProscan**: version 5.22-61.0, with parameters -goterms, --pathways and using an –iprlookup; **25) BUSCO**: version 1.22; **26) GMAP**: version 2017-06-20, parameters: -D, -S, -f; **27) LAST**: version 914.

## Data Records

This Whole Genome Shotgun project has been deposited at DDBJ/ENA/GenBank (Data Citation 1). Raw read files are available at NCBI Sequence Read Archive (Data Citation 2).

## Technical Validation

### Assessing the completeness of the genome assembly and annotation

We used CEGMA^31^ and BUSCO^38^, tools that investigate the presence of highly conserved orthologous genes in a genome assembly, to perform a first evaluation of the quality of the cork oak draft genome. CEGMA contains a total of 248 core eukaryotic genes. BUSCO was run over the plant set, which contains a total of 956 orthologue groups, defining *Arabidopsis* as the model species for the gene prediction performed by Augustus within the BUSCO pipeline. The results showed that the vast majority of the core genes/orthologues was present in the genome assembly (98.8% CEGMA, 95.6% BUSCO), and with complete matches (98.0% CEGMA, 94.9% BUSCO), which evidenced a good quality of the draft genome, in particular of the gene content and transcriptome. The number of complete orthologs found to be duplicated using BUSCO was 130, which represented 13.6% of the total (14.3% of the complete orthologs), percentages that are lower than the ones determined for the valley^16^ and pedunculated^37^genomes (52 and 49%, respectively).

### Structural annotation validation with RNA-Seq data

Predicted gene models were evaluated by running Stringtie over the RNA-Seq libraries, using the Augustus annotation as the reference and more stringent settings for the identification of transcripts. High confidence tags were assigned to the reference genes for which there were assembled transcripts within the genome coordinates determined during the annotation step. A total of 33,658 predicted genes were confirmed as high confidence genes with the analysis of the RNA-Seq data, which represented 42.2% of the 79,752 predicted genes. It should be emphasized that this validation procedure was limited to the genes expressed in the five tissues for which RNA-Seq data was available. Hence, assuming that transcriptomic data derived from different cork oak tissues and organs will likely contain a distinct set of expressed genes, it is reasonable to assume that more genes would be validated, emphasizing the quality of the genome annotation produced.

### Read mapping against the cork oak draft genome

The availability of several sets of cork oak high-throughput sequence data, produced with different types of sequencing protocols and in diverse individuals, enabled investigating the performance of the draft genome sequence in read mapping experiments, since these represent the analysis step expected to be most commonly used by other researchers. The available reads were retrieved from NCBI’s Sequence Read Archive (SRA) and were derived from RNA-Seq (generated in the 454 and Illumina platforms) and small RNA-Seq experiments, including the response of cork oak roots to drought^3^, the transcriptomic analysis of male and female flowers^4^, the comparison of good and bad quality cork samples^39^, the response of oak roots to the establishment of ectomycorrhizal symbiosis^2^, the dynamics of cork oak somatic embryogenesis (unpublished results) and miRNA profiling in leaf and cork tissues^40^. Additionally, the RNA-Seq reads derived from the five cork oak tissues were also used, even though this read dataset was derived from samples collected in the tree (HL8) used for genome sequencing. The Illumina RNA-Seq reads were mapped to the draft genome using Star, while GMAP^41^ and BWA-mem were used to map the RNA-Seq 454 and small RNA-Seq reads, respectively. Since the Illumina reads were produced using a paired-end sequencing protocol, the number of mapped reads was defined as the reads that were mapped as proper pairs, following the information specific to each PE library, while for the 454 reads only the reads that mapped to a unique genome location were considered. The percentage of mapped reads ranged from 65.6% to 89.6%, for the SRR1012034 and SRX2239662datasets, respectively (Table 9).

### Comparison with other *Quercus* genomes

Recently, the draft genome sequences for two other species of the *Quercus* genus were released, which included pedunculated oak (*Quercus robur*)^37^ and valley oak (*Quercus lobata*)^16^. A comparison of the assembly metrics of the cork oak draft genome with these two genomes indicated a similar number of scaffolds longer than 2 kb (15,058 cork oak; 17,910 pedunculate oak; 18,512 valley oak), even though the total genome assembly included in these scaffolds differed between species (941.0 Mb cork oak; 1.34 Gb pedunculated oak; 1.15 Gb valley oak). The N50 scaffold size of the cork oak assembly was 465.2 kb, while for pedunculated oak and valley oak the N50 values were 260 kb and 278.1 kb, respectively. Moreover, the heterozygosity estimates for cork oak (1.62%) and valley oak (1.25%) indicate that *Quercus* genomes indeed display high levels of heterozygosity. Additionally, the similarity level between the cork oak and the other two *Quercus* genomes, defined as the percentage of the cork oak genome that aligned with the other *Quercus* genomes, was determined aligning the valley and pedunculated oak genomes to the cork oak genome, using LAST^42^. The results showed that at the nucleotide level the similarity between the cork oak and valley and pedunculated oak was estimated to be 36.7 and 58.7%, respectively.

As this analysis did not discriminate between gene and non-gene space, a similar analysis was performed using the set of BUSCO orthologs found in each of the *Quercus* species. BUSCO was run with the same parameters described before over the valley and pedunculated oak genomes, in order to obtain the set of orthologs identified within their genomes. For this analysis two sets of orthologs were used, which included the orthologs found in common between cork oak and valley oak and the ones found in common between cork oak and pedunculated oak. In both cases, only the orthologs defined as “Complete” and “Duplicated” were used. As the number of copies of the orthologs between the three species was different only the largest set for each ortholog identifier was considered for the analysis. The final set for the *Quercus suber* vs. *Quercus lobata* comparison contained a total of 880 orthologs, while the set of *Quercus suber* vs. *Quercus robur* contained a total of 890 orthologs. After aligning the valley and pedunculated oak orthologs against the ones from cork oak, the similarity at the nucleotide level was estimated to be 78.8% and 81.8%, respectively. Additionally, the orthologs in common between valley and pedunculated oaks (895) were also aligned using the valley oak as a reference, which resulted in an estimated similarity of 85.3%. Previously, the identity between the valley and pedunculated oak genomes was determined to be 93.1% (ref. 16). When focusing on the set of conserved orthologs identified by BUSCO, the three *Quercus* species display a high percentage of similarity between them. While the valley and pedunculated oaks present more similarity between them, cork oak is genetically more distant from these two species.

Even though the unique genome characteristics of each species must be considered, the differences observed on the genome assembly metrics were not substantial and may be related to the sequence datasets and assembly strategies followed in each study. A more comprehensive comparison will be possible once higher quality genome assemblies for these, and possibly other, *Quercus* species become available.

### Final considerations

These results obtained in this study fully validate the usefulness of the assembled cork oak draft genome for several different types of genomic studies in this species. These resources will help cork oak becoming a useful model for studying *Quercus* species, for instance for genome evolution studies, and it will also greatly enhance the development and execution of current and novel research lines tackling the main concerns regarding cork oak and the cork industry. Additional work, carried by our research team, is currently underway, in order to develop an improved version of the cork oak genome and generate a detailed molecular characterization of the mechanisms underlying cork formation.

## Additional information

**How to cite this article:** Ramos, A. M. *et al.* The draft genome sequence of cork oak. *Sci. Data* 5:180069 doi: 10.1038/sdata.2018.69 (2017).

**Publisher’s note:** Springer Nature remains neutral with regard to jurisdictional claims in published maps and institutional affiliations.

## Supplementary Material



## Figures and Tables

**Table 1:** Microsatellites used for genotyping the 28 cork oak individuals for selection of the tree used in genome sequencing.

**Reference**			**Size (bp)**	
	**Locus**	**Motif**	**Expected**	**Observed**
Ueno and Tsumura^7^	QrOST1 (DN950446)	(AG)_19_	149–171	134–152
	QpD12 (CR627959)	(GCA)_7_	243–251	240–254
Steinkellner *et al*.^8^	QpZAG110	(AG)_15_	206–262	200–260
	QpZAG9	(AG)_12_	182–210	223–249
	QpZAG15	(AG)_23_	108–152	101–135
	QpZAG36	(AG)_19_	210–236	205–231
	QpZAG46	(AG)_13_	190–222	180–200
Kampfer *et al.*	QrZAG20	(TC)_18_	160–200	161–179
	QrZAG7	(TC)_17_	115–153	115–133
	QrZAG11	(TC)_22_	238–263	255–281
Dow *et al*. ^10^	MSQ4	(AG)_17_	203–227	186–218
	MSQ13	(TC)_11_	222–246	218–230
Isagi and Suhandono^11^	QM3-50/QM50-3M	Composite	253	273–289
Sebastiani *et al.*^12^	Cmcs1	(AT)_7_	104–108	104–123
CEBAL	CB01	(TC)2	-	88–106
INIAV	D8	(CA)_20_	141–151	139–155
For each microsatellite the motif, as well as the expected and observed sizes, are indicated.				

**Table 2:** Illumina DNA sequencing metrics, before and after preprocessing.

**Library Type**	**Insert Size (bp)**	**Illumina Sequencer**	**Read Length**	**Number libraries**	**Original Number of Reads**	**Reads Kept After Pre-processing**	**Percentage reads kept**
Paired-End	170	HiSeq 2000	100	3	983,306,498	833,615,836	84.8
	300	HiSeq X Ten	150	6	5,648,976,124	4,637,949,574	82.1
	500	HiSeq 2000	100	3	924,063,928	750,835,264	81.3
	800	HiSeq 2000	100	3	622,761,676	467,933,516	75.1
Mate-Pair	2000	HiSeq 2000	49	6	1,419,111,502	1,166,743,086	82.2
	5000	HiSeq 2000	49	3	501,967,462	404,751,694	80.6
	10000	HiSeq 4000	49	3	202,568,340	124,339,922	61.4
	20000	HiSeq 4000	49	3	258,232,918	157,943,444	61.2
When necessary, the reads were trimmed, using Sickle’s sliding window approach, to a minimum length of 120, 80 and 40 nucleotides, for the PE150, PE100 and MP libraries, respectively. The minimum quality over the set window size for each library type was 20.							

**Table 3:** NCBI Reference Sequence numbers for the chloroplast and mitochondrion genomes used in the preprocessing step.

**Chloroplast genomes**		**Mitochondrion genomes**	
NC_000932.1	*Arabidopsis thaliana*	NC_001284.2	*Arabidopsis thaliana*
NC_014674.1	*Castanea mollissima*	NC_014043.1	*Citrullus lanatus*
NC_023801.1	*Castanopsis echinocarpa*	NC_016005.1	*Cucumis sativus*
NC_009143.1	*Populus trichocarpa*	NC_014050.1	*Cucurbita pepo*
NC_026790.1	*Quercus aliena*	NC_016743.2	*Lotus japonicus* strain MG-20
NC_026913.1	*Quercus aquifolioides*	NC_018554.1	*Malus x domestica*
NC_026907.1	*Quercus spinosa*	NC_016742.1	*Millettia pinnata*
NC_023959.1	*Trigonobalanus doichangensis*	NC_028096.1	*Populus tremula*
A total of 16 genomes were used, eight from each organelle, for a total of 15 distinct plant species.			

**Table 4:** Illumina RNA sequencing metrics, before and after preprocessing.

**Plant Tissue**	**Number of libraries**	**Original number of reads**	**Reads kept after pre-processing**	**Percentage of reads kept**
Pollen	1	197,725,257	192,418,402	97.3
Leaf	2	299,960,018	280,640,162	93.6
Xylem	2	361,255,569	338,218,694	93.6
Inner bark	2	311,378,053	291,162,581	93.5
Phellem	2	360,128,704	335,696,318	93.2
The reads were trimmed, when required, to a minimum length of 80 nucleotides, using Sickle^13^ and a minimum quality of 20.				

**Table 5:** Metrics after integration of the paired-end assemblies generated during the process of producing the draft cork oak genome.

**Size range (bp)**	**Number of contigs**	**Total length (bp)**	**Percentage of assembly**
≥ 1,000	168,041	939,042,321	100
≥ 2,000	126,359	877,345,132	93.4
≥ 3,000	97,522	806,202,144	85.9
≥ 4,000	77,322	736,006,133	78.4
≥ 5,000	62,344	668,885,815	71.2
≥ 6,000	50,853	605,984,813	64.5
≥ 7,000	41,920	548,098,241	58.4
≥ 8,000	34,715	494,194,371	52.6
≥ 10,000	24,215	400,472,101	42.6
≥ 12,500	15,612	304,434,896	32.4
≥ 25,000	2,384	84,635,745	9.0
≥ 50,000	238	16,928,971	1.8
≥ 75,000	74	7,341,458	0.8
≥ 100,000	30	3,667,156	0.4
The integration of the two paired-end assemblies was performed using GARM^20^. The number of contigs for different size ranges, as well as the total length and the percentage of the assembly for each size range, are indicated.			

**Table 6:** Assembly metrics for the draft cork oak genome.

**Size range (bp)**	**Number of scaffolds**	**Total length (bp)**	**Percentage of genome assembly**
≥ 1,000	23,344	953,298,672	100
≥ 2,000	15,058	940,958,981	98.7
≥ 2,500	11,728	933,487,254	97.9
≥ 10,000	4,730	901,545,014	94.6
≥ 50,000	2,449	855,598,422	89.8
≥ 100,000	2,022	823,714,113	86.4
≥ 250,000	1,207	687,189,587	72.1
≥ 500,000	539	445,055,144	46.7
≥ 750,000	249	268,162,306	28.1
≥ 1,000,000	119	157,303,135	16.5
≥ 1,500,000	28	48,679,102	5.1
≥ 2,000,000	5	10,685,375	1.1
The number of scaffolds is indicated for different size ranges, which also include the total length and the percentage of the genome assembly for each size range.			

**Table 7:** Functional annotation results of the 83,814 predicted cork oak transcripts.

**Database**	**Number of annotated transcripts**	**Percentage**
NCBI-nr-plants	56,496	67.4
SwissProt	46,602	55.6
Eggnog Viridiplantae	49,518	59.1
InterPro	69,218	82.6
The results obtained using four different databases are presented, including the percentage of transcripts functionally annotated.		

**Table 8:** Results obtained with RepeatMasker for the cork oak draft genome.

	**Number of Elements**	**Length Occupied (bp)**	**Percentage of Sequence (%)**
Retroelements	96,642	72,329,781	7.59
SINEs:	596	71,956	0.01
Penelope	3	438	0.00
LINEs:	24,465	12,440,942	1.31
CRE/SLACS	35	1,803	0.00
L2/CR1/Rex	0	0	0.00
R1/LOA/Jockey	0	0	0.00
R2/R4/NeSL	0	0	0.00
RTE/Bov-B	2,551	734,240	0.08
L1/CIN4	21,858	11,703,615	1.23
LTR elements:	71,581	59,816,883	6.27
BEL/Pao	0	0	0.00
Ty1/Copia	31,107	25,406,691	2.67
Gypsy/DIRS1	36,363	32,397,852	3.40
Retroviral	0	0	0.00
DNA transposons	32,632	7,066,773	0.74
Hobo-Activator	12,038	3,473,326	0.36
Tc1-IS630-Pogo	280	28,138	0.00
En-Spm	0	0	0.00
MuDR-IS905	0	0	0.00
PiggyBac	0	0	0.00
Tourist/Harbinger	2,785	708,244	0.07
Other (Mirage P-element, P-element, Transib)	0	0	0.00
Rolling-circles	0	0	0.00
Unclassified	4,448	1,927,308	0.20
Total interspersed repeats		81,323,862	8.53
Small RNA	1,303	233,092	0.02
Satellites	321	36,450	0.00
Simple repeats	700,496	25,912,290	2.72
Low complexity	127,202	6,580,532	0.69
The whole set of scaffolds (23,344) was used in the run, with a total sequence length of 953.3 Mb.			

**Table 9:** Summary statistics for the mapping of several read datasets against the cork oak draft genome.

**Study and data type**	**SRA accession number**	**Total number of reads**	**Number of mapped reads**	**Percentage of mapped reads**
Magalhães *et al*.^3^ (454)				
Medium drought	SRR1812375	587,184	439,362	74.8
Severe drought	SRR1812376	473,117	342,671	72.4
Well-watered	SRR1812377	645,761	484,889	75.1
Rocheta *et al*.^4^ (454)				
Male flower	SRR1609152	659,399	493,298	74.8
Female flower	SRR1609153	535,665	401,025	74.9
Teixeira *et al*.^39^ (454)				
Good cork quality	SRR1009171	573,548	409,169	71.3
Bad cork quality	SRR1009172	600,102	417,493	69.6
Sebastiana *et al*.^2^ (454)				
ECM roots	SRR1012033	1,159,845	769,423	66.3
Non-symbiotic roots	SRR1012034	969,271	636,048	65.6
Somatic embryogenesis (Illumina PE)				
Embryo globular stage	SRX2239661	71,706,998	62,998,568	87.9
Embryo heart/torpedo stage	SRX2239662	71,964,732	64,487,926	89.6
Embryo immature cotyledonary stage	SRX2239663	84,482,546	73,969,188	87.6
Embryo mature cotyledonary stage	SRX2239664	84,022,498	73,715,150	87.7
Chaves *et al*.^40^ (Illumina SE)				
Leaf	SRR988108	16,838,439	13,900,006	82.5
Cork	SRR988109	9,333,712	7,223,415	77.4
HL8 RNA-Seq (Illumina PE)				
Pollen	SRR5986741	192,418,402	161,872,000	84.1
Leaf	SRR5986739	299,960,018	245,400,286	81.8
Xylem	SRR5986738	361,255,569	296,903,800	82.2
Inner bark	SRR5986740	311,378,053	251,537,756	80.8
Phellem	SRR5986737	353,687,405	285,006,172	80.6
The reads were downloaded from NCBI’s Sequence Read Archive and mapped to the draft genome using BWA-mem.				
